# Hot liver on 18F-FDG PET/CT imaging—a quandary for oncoradiologists in South Asia

**DOI:** 10.1259/bjrcr.20190097

**Published:** 2020-09-29

**Authors:** Vijayakumary B, Ramachandran C, Bharath V M, Madhu Muralee, Jubie Raj

**Affiliations:** 1 Department of Radiology, Regional cancer centre, Thiruvananthapuram, Kerala, India; 2 Department of Surgical oncology, Regional cancer centre, Thiruvananthapuram, Kerala, India

## Abstract

A 56-year-old female presented with intermittent hemoptysis and was diagnosed with lung cancer. ^18^F-fluorodeoxyglucose positron emission tomography/CT for staging revealed hypermetabolic liver (hot liver), uptake in the mediastinal lymph nodes and reduced uptake in the kidneys. Unexpectedly, liver biopsy findings were consistent with tuberculous infection. Following the intensive phase of antituberculous treatment, repeat CT revealed significant resolution of the mediastinal lymph nodes making the lung cancer Stage 1 (T1 lesion). She underwent left lower lobectomy as a definitive surgical treatment. Positron emissiontomography/CT scan in this patient was considered to be a hepatic superscan since it revealed a hot liver.

## Clinical presentation

A 56-year-old female presented with intermittent hemoptysis. Elevated erythrocyte sedimentation rate (ESR) of 90 mm/h was the only positive laboratory workup finding.

## Investigations and imaging findings

**Figure 1.**
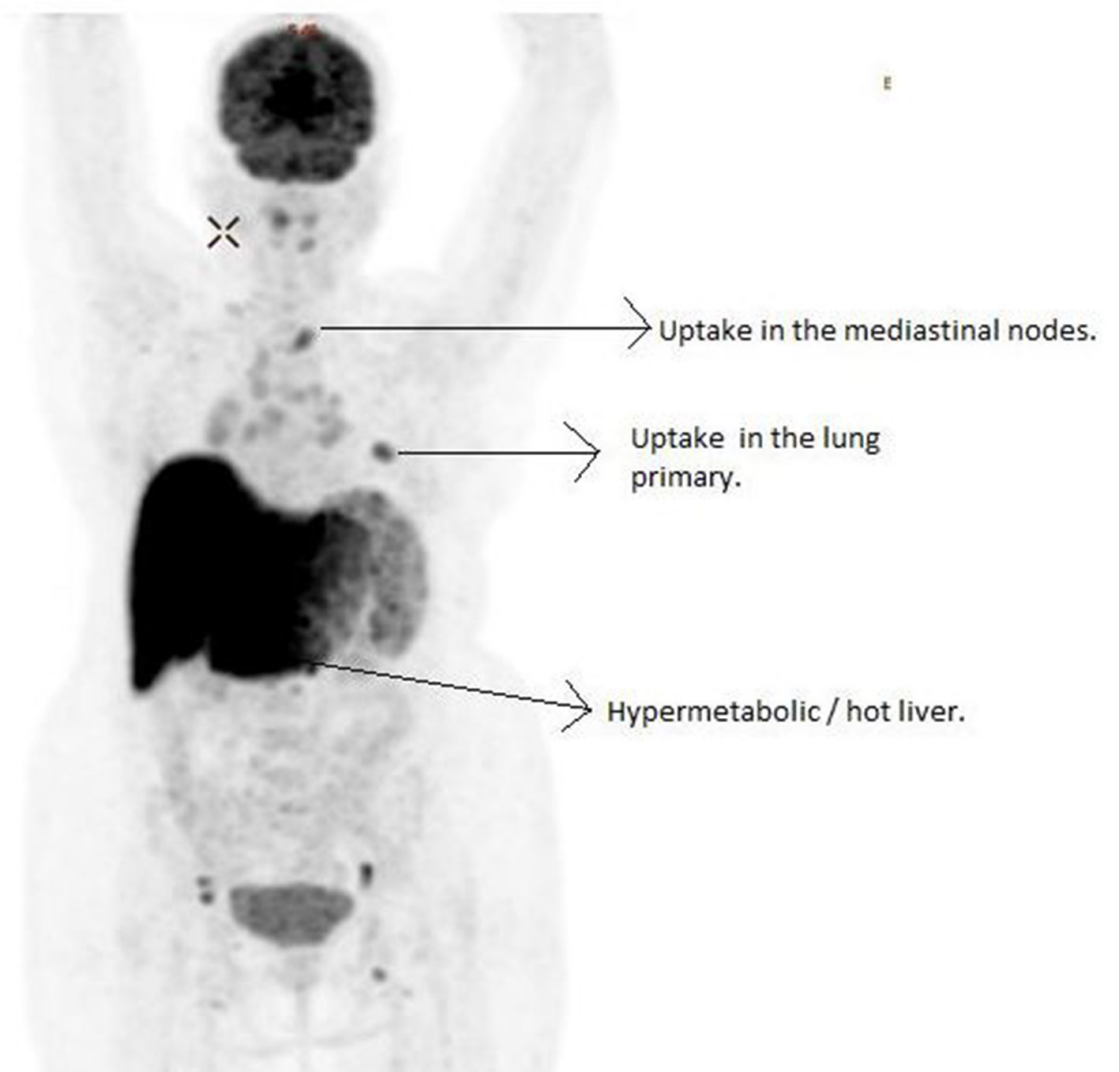
PET image (obtained 1 hr after administration of 5.2 mCi of ^18^F-FDG) showing focus of uptake in the left lung lower zone, corresponding to the lung primary. Uptake is noted in the mediastinal lymphnodes also. The liver is appearing enlarged with markedly increased uptake, s/o hypermetabolic liver/hot liver. The uptake in the brain and collecting system are noted, even though to a lesser extent. FDG, fluorodeoxyglucose; PET, positron emission tomography.

**Figure 2.**
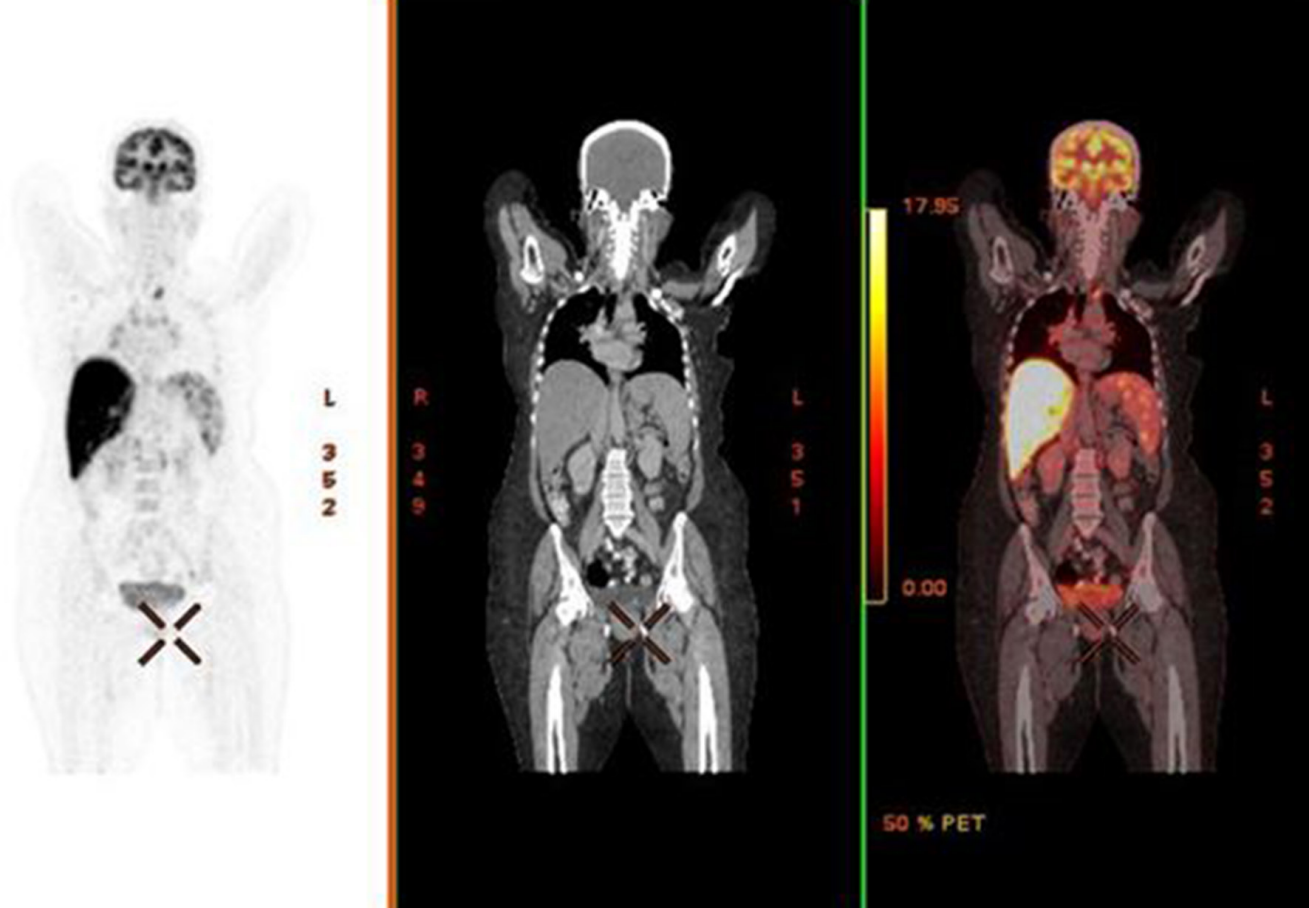
Coronal PET, CT and corresponding fused PET/CT image showing hypermetabolic/hot liver. PET image obtained one hour after administration of 5.2 mCi of ^18^F-FDG. CT done without administering intravenous contrast. FDG, fluorodeoxyglucose; PET, positron emission tomography.

**Figure 3.**
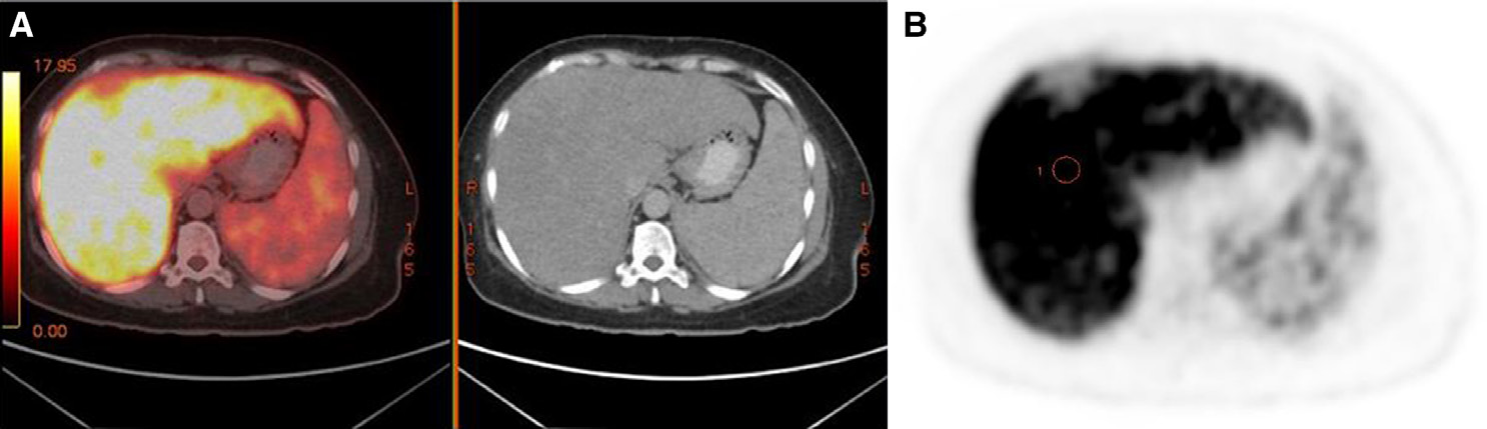
(a) Axial fused PET/CT image and CT image (obtained one hour after administration of 5.2 mCi of ^18^F-FDG) show intense uptake in the liver. (b) Axial PET image shows intense uptake in the liver. FDG, fluorodeoxyglucose; PET, positron emission tomography.

A chest X-ray followed by CT revealed a spiculated lesion in the left lower lobe lung of approximately 2.5 cm size. Imaged biopsy of which diagnosed it as non-small cell carcinoma of the lung (moderately differentiated adenocarcinoma). CT also revealed few necrotic mediastinal lymph nodes, without any calcification in the left paratracheal location and mild hepatomegaly without any focal lesions. ^18^F-FDG PET/CT (^F^18-fluorodeoxyglucose positron emission tomography/CT) for staging revealed diffuse and extensive uptake in the liver (hypermetabolic liver or rather hot liver) and also by the mediastinal lymph nodes (Figures 1–3). The uptake in the kidneys was considerably less. We considered the possibility of hot/hypermetabolic liver due to massive metastasis and metastatic mediastinal lymphadenopathy.

The patient required either EBUS-TBNA (endobronchial ultrasound-transbronchial needle aspiration) or mediastinoscopic biopsy of lymph nodes or liver biopsy to confirm metastases in the respective sites (to rule out false positivity of PET/CT). We performed ultrasound-guided liver biopsy in this patient, because of the ready availability and ease of this procedure compared to the others.

**Figure 4.**
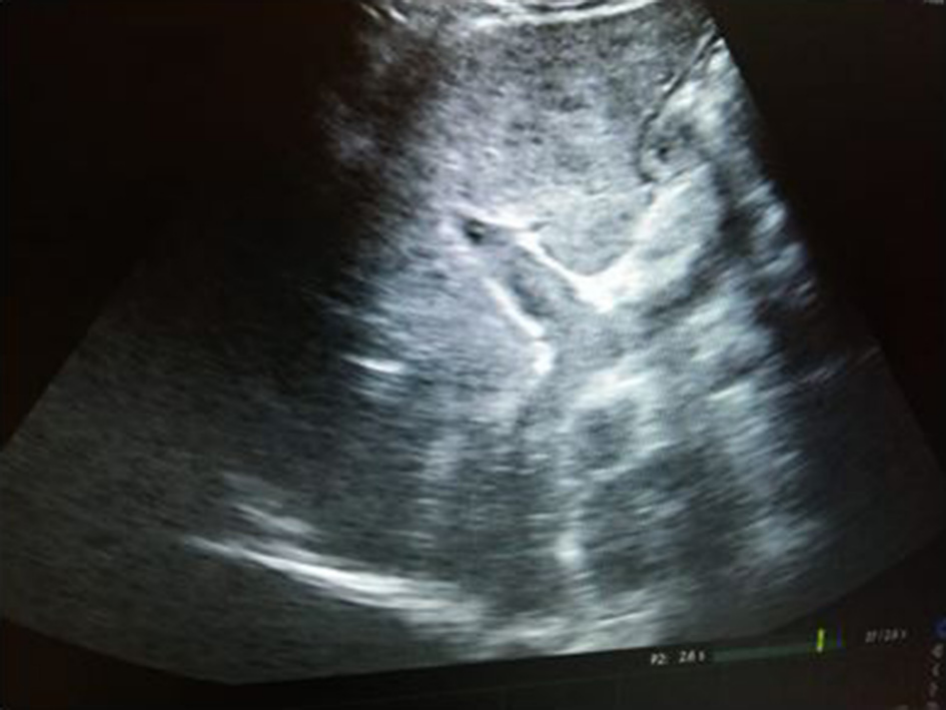
Ultrasound image of the right lobe of liver shows heterogeneous echotexture. Biopsy was taken from the heterogeneous appearing area of the right lobe of liver under ultrasound guidance.

**Figure 5.**
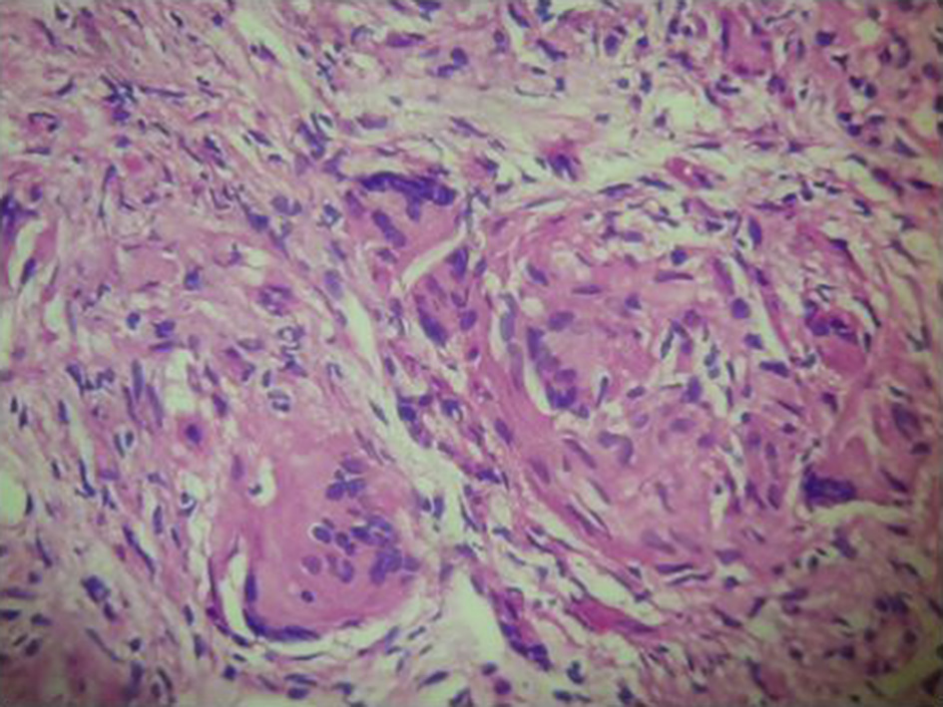
Liver biopsy shows infiltration of the liver parenchyma with multiple inflammatory cells. There is evidence of granuloma formation.

Ultrasound performed showed coarsening of liver parenchyma (Figure 4), which is not specific for any particular pathology. Biopsy from a relatively heterogeneous appearing area of the right lobe of the liver revealed plenty of inflammatory cells infiltrating liver parenchyma with granuloma formation (Figure 5). Acid-fast smear done was positive, consistent with active tuberculous infection (miliary hepatic tuberculosis).

## Treatment

After a discussion with MDT (multidisciplinary tumor board), the patient was started on antituberculous treatment. Repeat CT (as the patient could not afford a repeat PET/CT due to financial constraints) after 2 months of the intensive phase of antituberculous drug treatment, revealed significant resolution of mediastinal lymph nodes which confirms the false positivity of mediastinal lymphadenopathy. The lung cancer was thus staged as T1 lesion, instead of Stage IV disease (AJCC TNM staging eighth edition).

**Figure 6.**
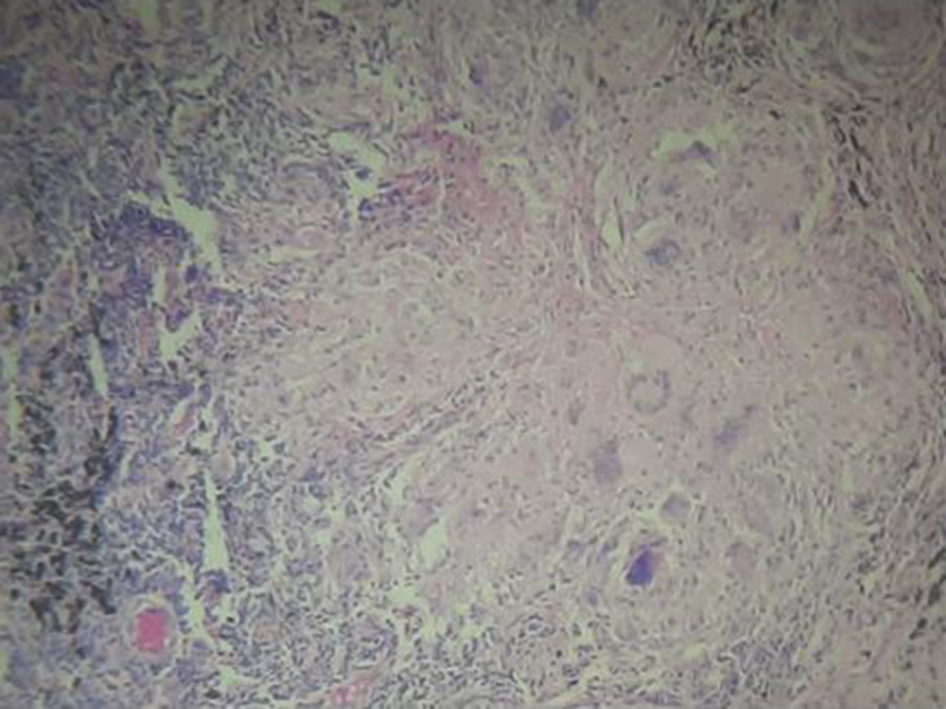
Lung, left lower lobectomy specimen showing multiple granulomas within the lung parenchyma. Towards the left side of this image, there are malignant cells, corresponding to the lung primary.

**Figure 7.**
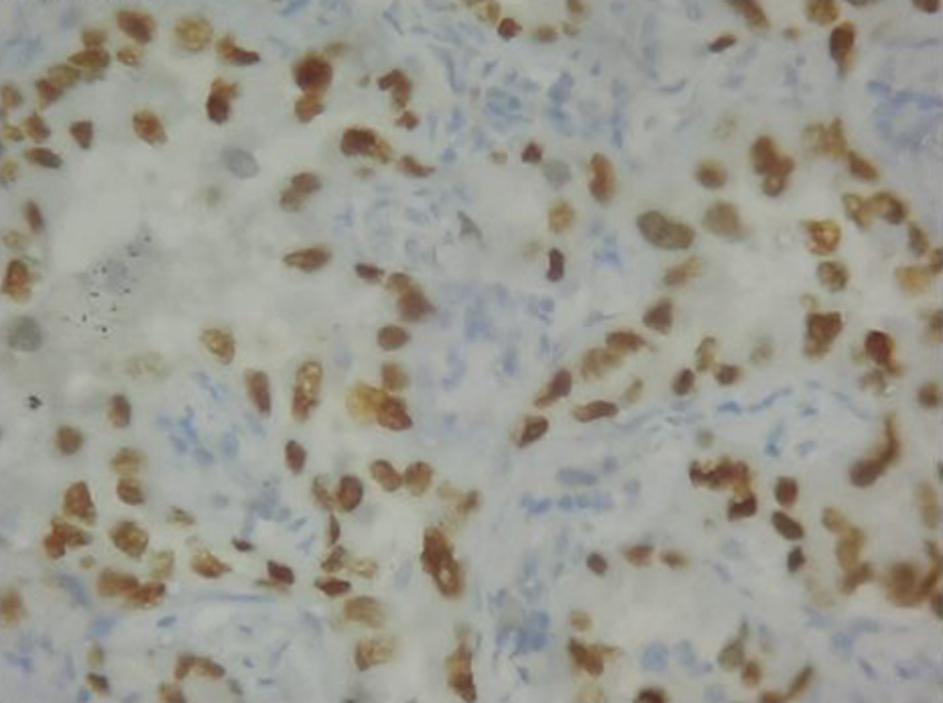
Left lung lower lobectomy specimen showing TTF-1 positivity. TTF-1, thyroid transcription factor-1.

She underwent left lower lobectomy as a definite surgical treatment. Histopathological examination of the left lower lobectomy specimen showed moderately differentiated adenocarcinoma of the lung with a predominant lepidic pattern. Adjacent lung parenchyma showed multiple granulomas (Figure 6). Immunohistochemistry done showed thyroid transcription factor-1 positivity (Figure 7). No lymph nodes were positive for malignancy. She later completed her antituberculous treatment. No further treatment was required for her lung malignancy since the lesion was only 2.5 cm and no positive lymph nodes were obtained.

## Outcome and follow-up

She was declared cured of her tuberculosis on completing antituberculous treatment. Follow-up imaging after 2 years showed disease-free status.

## Discussion

Superscan is classically a ‘super bone scan’ of skeletal scintigraphy.^1^ A similar term is also employed in ^18^F-FDG PET/CT imaging.

Superscan^2^ in ^18^F-FDG PET/CT is described as sequestration of the radiopharmaceutical in an organ or an organ system with reduction or complete absence in the activity detected in those organs which are normally associated with significant physiologic uptake of ^18^F-FDG. The organs showing normal physiological uptake are kidneys, collecting systems, brain, and heart. Hepatic superscan/hot liver is characterized by intense uptake in the liver with suppression of the background physiological activity.

Superscan appearance in the liver in ^18^F-FDG PET/CT is usually associated with malignancy, like massive liver metastases (breast primary), primary hepatic lymphoma, diffuse angiosarcoma of the liver, secondary lymphomatous or leukemic involvement of liver etc. Usually infective etiologies like hepatic tuberculosis,^3^ Q fever hepatitis will cause increased uptake in the liver without significant suppression of the background physiological activity.^2^ Metastases from lung cancer rarely cause superscan appearance.^4^ Very rarely, even infective etiologies can cause superscan appearance, which is the situation in our case.

There is an increasing incidence of typical and atypical forms of tuberculosis, especially in developing countries, partly due to the development of drug resistance and partly due to increasing HIV incidence. In a developing country like India, where tuberculosis is endemic, the possibility of the same should be born in mind while considering the differentials of diffuse hepatic involvement. Biopsy proven lung cancer misleads us to believe that hepatic involvement is probably metastatic. However, decision to go ahead with liver biopsy helped us to understand the false positivity of hepatic superscan, detect and treat a potentially fatal infection and perform definitive and curative surgery for lung cancer.

## Conclusion

It is always safe to consider the possibility of miliary hepatic tuberculosis, whenever we are dealing with a case of hypermetabolic liver, especially in a tuberculosis endemic area. The same had helped us to offer definitive and curative treatment to our patient.

## Learning points

- Even in the background of proven malignancy, the infective processes like tuberculosis (Koch’s) should be considered as a differential diagnosis for hypermetabolic PET/CT foci, especially in endemic areas like South Asia.
- In cases of diffuse hepatic involvement, liver biopsy when performed under ultrasound guidance from a relatively heterogeneous appearing area gives a good yield.
- Hepatic superscan can have a false-positive interpretation for metastases.
- Tuberculosis in our case was either subclinical/initial presentation of intermittent hemoptysis was due to pulmonary tuberculosis rather than due to malignancy.
